# A Diagnostically Challenging Case of Recurrent Encephalitis With Impending Herniation

**DOI:** 10.7759/cureus.67599

**Published:** 2024-08-23

**Authors:** Lina Okar, Sandra Samuel, Divya Singh, Francesca Pastrana, Fajun Wang

**Affiliations:** 1 Neurology, Saint Louis University School of Medicine, St. Louis, USA; 2 Medicine, Saint Louis University School of Medicine, St. Louis, USA; 3 Neurology, St. Louis University, St. Louis, USA; 4 Neurology, Mercy Hospital South, St. Louis, USA

**Keywords:** encephalitis, seizure, herpes simplex virus encephalitis, relapse encephalitis, acute encephalitis

## Abstract

Encephalitis is characterized by inflammation of the brain parenchyma with typical presenting symptoms of altered mental status and seizures. However, diagnostic workup is complex given the multitude of possible etiologies for encephalitis. Further, recurrence of encephalitis is rare, and understanding its risk factors, mechanisms, prognosis, and optimal treatment remains incomplete. Here, we present the case of a 69-year-old woman admitted to our hospital with altered mental status who was diagnosed with encephalitis based on clinical and imaging findings. This case highlights the diagnostic approaches required to obtain the final diagnosis and the treatment plan that resulted in the patient’s eventual return to baseline and functional independence.

## Introduction

Viral encephalitis is the most common etiology for encephalitis in adults. Herpes simplex virus (HSV-1) virus is the most common pathogen affecting 54% of the United States population between the ages of 14 and 49 [1]. HSV polymerase chain reaction (PCR) has more than 90% sensitivity and specificity and is considered the test of choice for diagnosis [2]. It could be falsely negative early in the disease (<3 days) or late (>14 days); therefore, acyclovir administration is advocated for high clinical suspicion. Additionally, a previous paper reported a case of negative HSV PCR 10 days after symptoms onset [3]. HSV encephalitis recurrence is uncommon in adults with fewer cases reported with negative PCR [4]. Data regarding risk factors, mechanisms, prognosis, and duration of therapy are not consistent in the literature [5].

Here, we present a challenging case of a 69-year-old woman who presented with severe recurrent viral encephalitis and an impending herniation. The patient managed a case of HSV encephalitis with a remarkable response to antiviral despite negative HSV PCR.

## Case presentation

**Figure 1:**
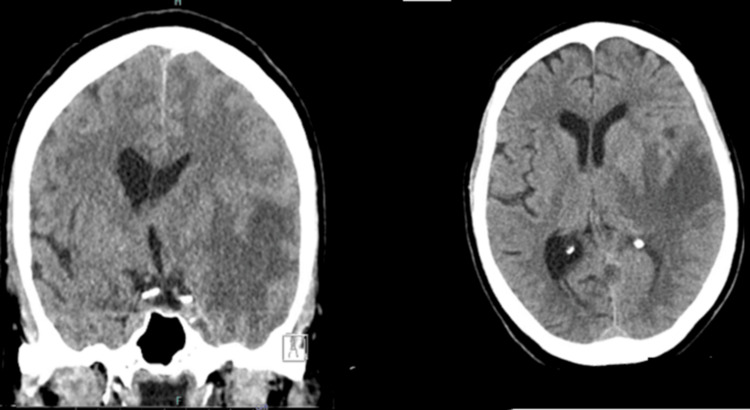
CT head without contrast on admission Findings of confluent area of edema with relative sparing of the cortex, predominantly involving the left temporal with extension into surroundings with mass effect on the left lateral ventricle with risk for herniation.

A 69-year-old female presented with altered mental status following witnessing seizure-like activity and was emergently intubated. Computed tomography (CT) head revealed an evolving left temporal lobe hypodensity with edema and impending uncal herniation (Figure 1). CT head and neck angiogram were unremarkable. Glasgow coma score (GCS) was 3 on arrival and neurological examination revealed intact brainstem reflexes and right-sided hemiplegia. Laboratory evaluation was unremarkable (Table 1). The patient was deemed not a candidate for thrombolysis given large-sized hypodensity. The patient was transferred to our hospital for further management. A review of history revealed a recent encephalitis affecting the right temporal lobe and diagnosis eight months prior with presentation of seizure and fever at which point she was empirically treated with acyclovir for 21 days with clinical recovery. The patient was also discharged on levetiracetam 750mg twice daily.

**Table 1:** Laboratory findings and initial lumbar puncture CSF results Abbreviations: WBC: White blood cells, RBCs: Red blood cells, HSV: Herpes simplex virus, PCR: Polymerase chain reaction; CMV: Cytomegalovirus

Test parameters (serum)	Results	Normal range
White blood cells (x10^9^/L)	9.1	4.4-10.7
Hemoglobin (g/dL)	11.3	12.0-15.6
Neutrophils (%)	77.5%	35.0-77%
Lymphocytes (10^3^/µL)	0.81	1.10-3.90
CSF study	Results	Normal range
Xanthochromia	Negative	NA
RBCs (x10^6^/L)	1,820	NA
WBCs (x10^6^/L)	20	0-5
Segmented cells %	73%	NA
Lymphocytes %	14%	NA
Protein (mg/dL)	109	14-45
Glucose	46	40-70
N-methyl-D-aspartate receptor antibody CSF	<1.1	NA
Varicella zoster virus antibodies	Negative	NA
HSV PCR	Not detected	NA
CMV, enterovirus, varicella	Negative	NA

**Figure 2:**
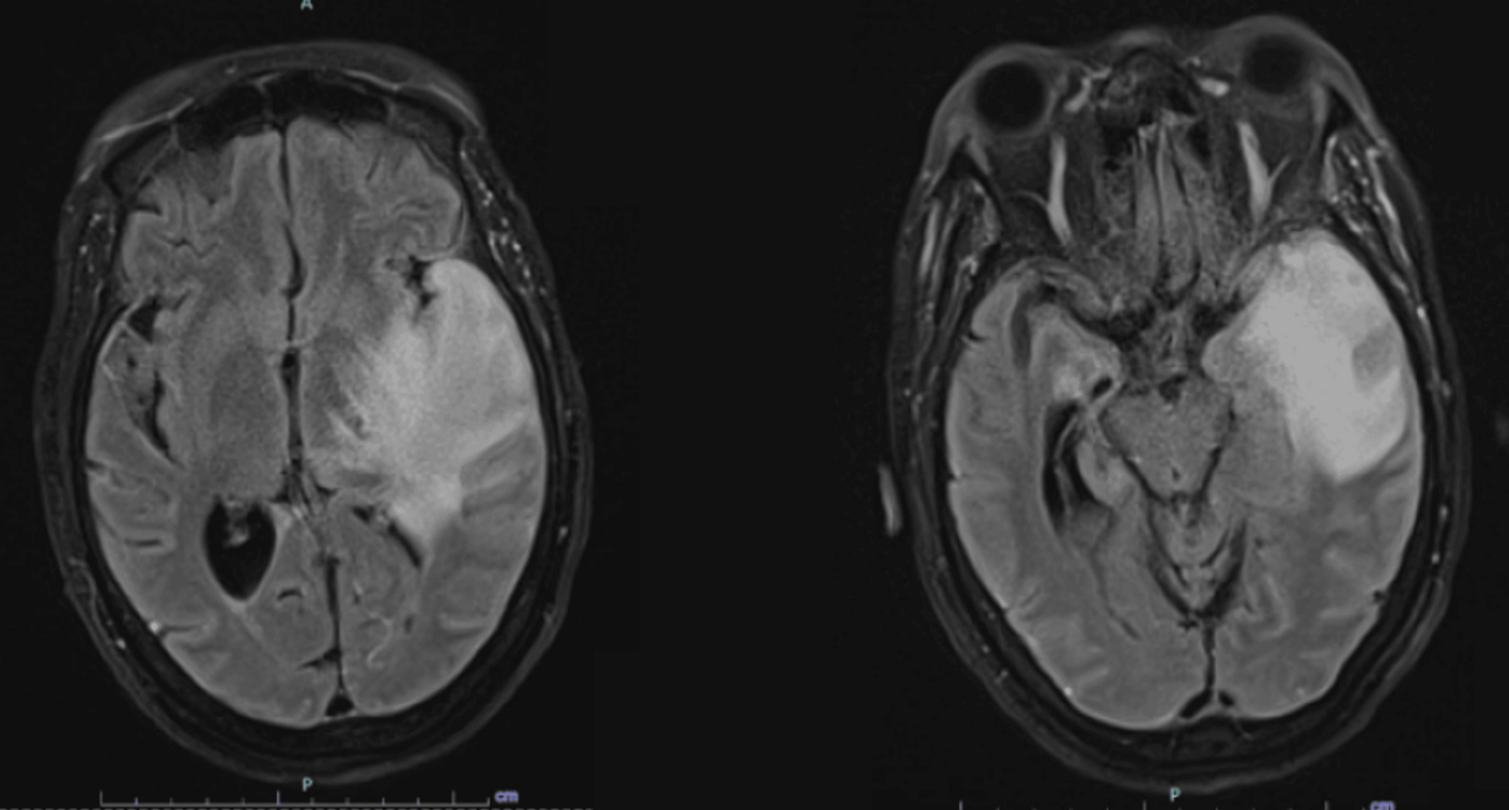
Initial MRI brain Findings of hyperintense areas on T2-weighted imaging, fluid-attenuated inversion recovery (FLAIR) accompanied by gadolinium enhancement on T1-weighted imaging in regions including temporal lobes, cingulate gyrus, orbitofrontal cortex, and insular cortex.

**Figure 3:**
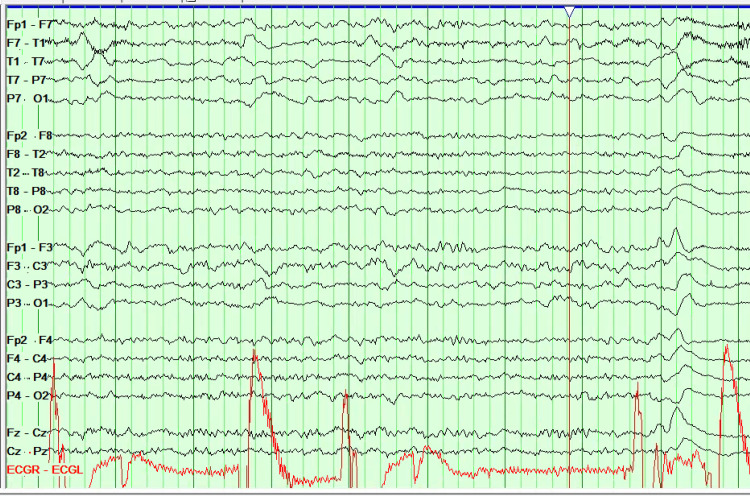
EEG findings showing prominent left Lateralized Periodic discharges (LDPs) over left temporal field

Given the presentation and CT findings, the patient was admitted to the Neurocritical Care Unit for initial concern of malignant MCA syndrome. However, brain magnetic resonance imaging (MRI) demonstrated a large area of confluent vasogenic edema in the left temporal lobe with leptomeningeal enhancement (Figure 2). Electroencephalography (EEG) showed left hemispheric lateralized periodic discharges and slowing (Figure 3).

**Figure 4:**
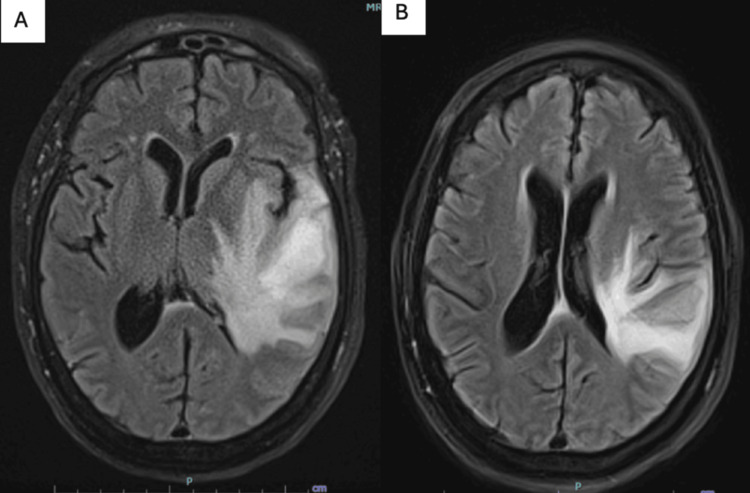
Repeated MRI imaging after two weeks (A) and three weeks (B) showing rapid improvement in the vasogenic edema on FLAIR FLAIR: fluid-attenuated inversion recovery

Levetiracetam was subsequently increased to 1000 mg twice daily. Differential diagnoses at this time included encephalitis due to viruses such as herpes simplex virus (HSV), cytomegalovirus (CMV), Epstein-Barr Virus (EBV), enterovirus, and varicella, autoimmune encephalitis and neoplasm. Due to the high mortality associated with untreated HSV encephalitis, empiric treatment with intravenous acyclovir was initiated. After discussions with neurosurgery, lumbar puncture (LP) was performed two days after initiation of antiviral therapy which revealed neutrophilic pleocytosis and negative HSV PCR and other viral tests (Table 1). In the meantime, her mentation and neurological exam continued to improve, she remained on the ventilator but was awake and alert, following commands and moving all extremities against resistance. Repeat MRI brain performed two and three weeks after admission (Figure 4) depicted decreasing edema and increased rim enhancement in the superior left temporal lobe consistent with evolving cerebritis with cystic encephalomalacia, as well as decreased leptomeningeal enhancement in the left parietotemporal lobe. She ultimately completed 14 days of IV acyclovir with significant clinical improvement despite repeat CSF studies repeat LP was again negative for HSV or autoimmune etiologies at days 7 and 12 post-admission but with lymphocyte predominance. The CSF study was concerning for infectious etiology given the pleocytosis and elevated protein. However, CSF protein was not elevated enough nor was any hypoglycorrhachia present to raise suspicion for bacterial etiology. The autoimmune process was unlikely given the negative panel and clinical improvement despite the lack of steroid treatment; the N-methyl-D-aspartate receptor (NMDAR) Antibody in CSF along with the full Mayoclinic autoimmune panel was negative. MR spectroscopy was performed and was inconclusive. Despite negative HSV PCR both in the past and on repeat LPs, clinical improvement with acyclovir therapy implies HSV encephalitis as the most probable diagnosis.

**Figure 5:**
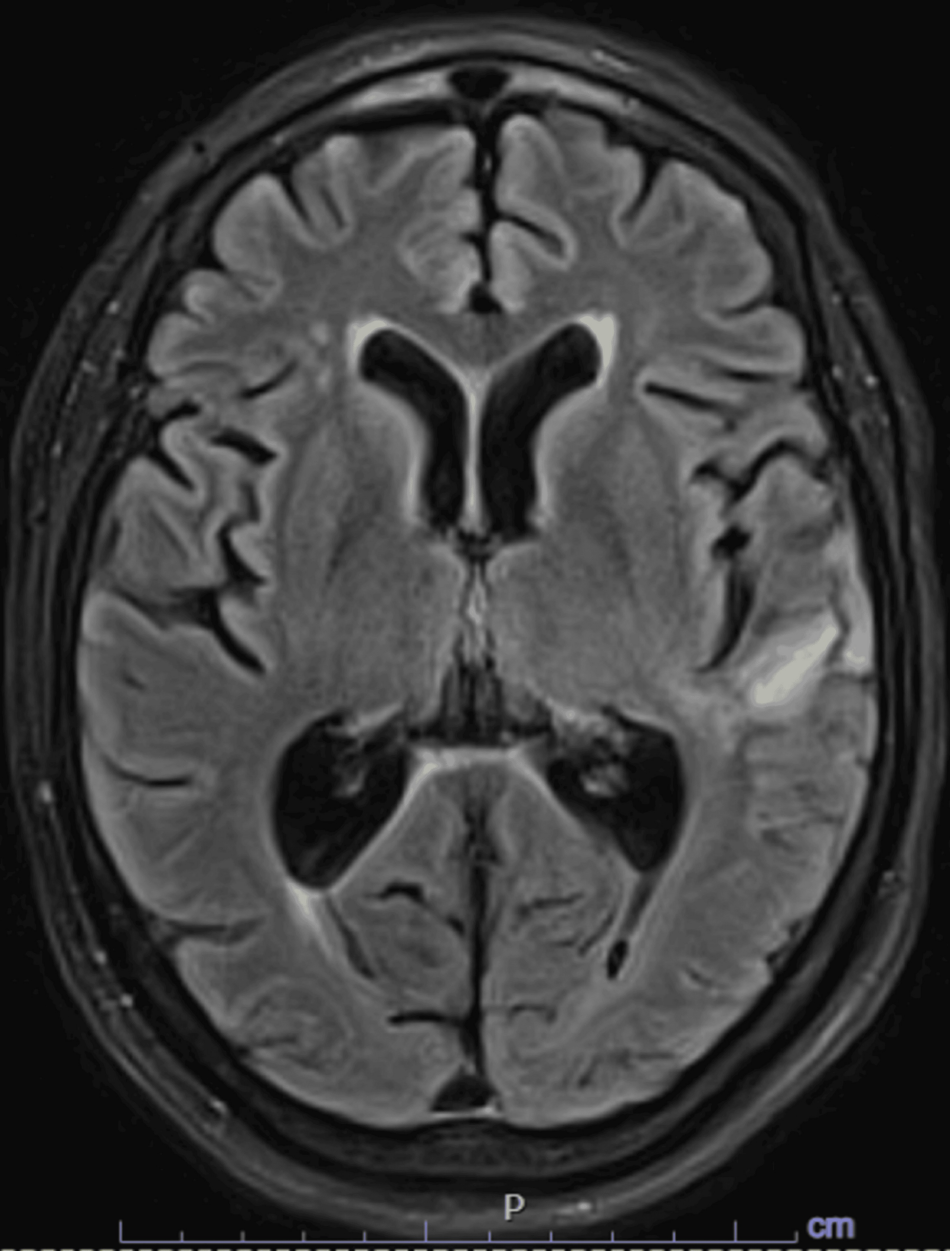
Follow-up MRI after three months showing significant improvement in left temporal lobe edema is noticed

Our patient was eventually discharged to a long-term care facility. In the outpatient clinic three months later, another repeat MRI revealed remarkable improvement (Figure 5), and she returned to baseline with only minor augmentative behavioral changes which resolved at six months follow up and the patient is back to her work performing independently in daily life activities.

## Discussion

Encephalitis, defined by inflammation within the brain parenchyma, presents a complex diagnostic and therapeutic challenge due to its diverse etiologies, including infectious and autoimmune causes [1]. The clinical spectrum of encephalitis is broad, ranging from focal neurological deficits and seizures to altered mental status, behavioral changes, status epilepticus, and even death [2]. Diagnosis relies heavily on history, laboratory, neuroimaging, and electrophysiological findings [3]. It should be suspected when acute neurological dysfunction presents alongside systemic symptoms, including fever, respiratory or gastrointestinal illness, or rash, typically within 24-72-hour prior [1]. In cases of high suspicion for viral encephalitis, administration of acyclovir before LP is recommended [3].

Various viruses have been described to cause encephalitis, including HSV types 1 and 2, non-polio enteroviruses, arboviruses, CMV, EBV, and Human Herpesvirus 6 (HHV-6). CSF tests in HSV encephalitis vary with the stage of the disease. Initially, CSF may be normal or show a predominance of polymorphonuclear cells, which later convert to lymphocytic predominance [4]. The gold standard for diagnosis of HSV encephalitis typically involves positive CSF HSV PCR, although other diagnostic clues may be present, such as pathognomonic findings on neuroimaging or EEG. On MRI, hyperintense areas may be observed on T2-weighted imaging, fluid-attenuated inversion recovery (FLAIR), or diffusion-weighted imaging, often accompanied by gadolinium enhancement on T1-weighted imaging in regions including temporal lobes, cingulate gyrus, orbitofrontal cortex, and insular cortex [3]. MRI is the gold standard imaging modality, with abnormalities detected in up to 90% of HSV encephalitis cases [5]. In our case, while the hypodensity on the CT head raised suspicion for acute infarction, the MRI obtained as part of the stroke work-up broadened the differential and led to change in management including LP. While the sensitivity and specificity of CSF HSV PCR during days two to 10 of symptom onset are 96% and 99%, respectively, our case was repeatedly negative [6]. EEG often reports non-specific slowing or periodic lateralizing epileptiform discharges in HSV encephalitis (1). If viral workup is negative, testing for antibodies that correlate with autoimmune encephalitis is important, such as antibodies against NMDAR or leucine-rich glioma-inactivated protein-(LGI-1). The fact that our patient responded both times to IV acyclovir will suggest HSV as the most likely cause. Immune-mediated encephalitis due to NMDA-Ab is also relatively common after an acute HSV encephalitis, yet our patient's anti-NMA Ab is negative.

Recurrence of viral encephalitis is rare in adults. Yamada et al. reported a case of relapsing HSV-1 encephalitis within five years of initial events in a 66-year-old immunocompetent gentleman with a poor prognosis and death due to respiratory infection [7]. Our patient was presumed to have viral encephalitis in her initial presentation based on clinical symptoms, MRI findings, and CSF profile and response to acyclovir. Her recurrence with similar clinical presentation but negative HSV PCR in multiple samples and again improved response to acyclovir implied the diagnosis of viral encephalitis. Prompt initiation of antiviral was also imperative in our case leading to significant improvement clinically and radiographically which has been estimated to reduce the mortality rate to 5%-20% from 70% when left untreated [8]. Herniation and increased intracranial pressure as consequences of HSV encephalitis have been described in the literature, with craniectomy sometimes considered a lifesaving procedure. However, early initiation of antiviral therapy remains the mainstay of treatment [9]. Our patient responded remarkably to antiviral therapy despite the severe initial presentation (GCS 3T) and impending herniation observed on imaging. An important consideration is the possibility of neoplasm. Piper et al. reported a case series of glioblastoma initially managed as viral encephalitis, with observed improvement following acyclovir administration, followed by the later diagnosis of glioblastoma [10]. In our patient, MR spectroscopy during admission was inconclusive and repeated MRI three months after discharge revealed evidence of injury resolution, which aligned with recovered infection.

It is not rare for a persistent inflammatory process to occur after viral encephalitis, but how it contributes to clinical symptoms is not determined [7]. In the case of HSV encephalitis, Suggested mechanisms for the recurrence in adults include inadequate antiviral therapy in the first event, viral reactivation, genetic predisposition, or immune-mediated [11-13].

## Conclusions

HSV encephalitis is a severe condition with high rates of both morbidity and mortality. HSV is known to establish latency in the human body, and reactivation of the virus has been linked to cases of recurrent encephalitis. Due to the challenges in early diagnosis, initiating empirical treatment with Acyclovir as soon as possible is critical to prevent serious complications. Our case describes a suspected recurrent severe viral encephalitis with impending herniation secondary to HSV in an otherwise immunocompetent patient with negative HSV PCR. This highlights the need to determine the diagnostic yield of PCR in recurrent viral encephalitis and identify risk factors, pattern of relapse, and ultimate duration of antiviral therapy. Additionally, the case reminds us of CSF alterations in the setting of viral encephalitis and the importance of early antiviral administration in decreasing mortality.
